# Supplementary material for: From sequence to enzyme mechanism using multi-label machine learning
Source: BMC Bioinformatics. 2014 May 19;15:150. doi: 10.1186/1471-2105-15-150 (PMC4229970; doi:10.1186/1471-2105-15-150)
Supplement: Additional file 2 — Java code of ml2db. Additional file ml2db_code.tar.gz contains the Java source code to run the multi-label machine learning experiments and save the results to database. The code’s Javadoc is included. [file 1471-2105-15-150-S2.zip › additional file 2/ml2db/utils/doc/index-files/index-13.html]

M-Index


JavaScript is disabled on your browser.


- Overview
- Package
- Class
- Use
- Tree
- Deprecated
- Index
- Help

- Prev Letter
- Next Letter

- Frames
- No Frames

- All Classes

A B C D E F G H I J K L M N O P Q R S T U V W X Y 


## M

m\_dbWriter - Variable in class uk.ac.standrews.utils.main.database.DbManager
:   the database recorder

m\_debug - Static variable in class uk.ac.standrews.utils.main.collectionutils.setutils.SupersetsManager


M\_DEBUG - Static variable in class uk.ac.standrews.utils.main.maputils.MapUtils


m\_debug - Static variable in class uk.ac.standrews.utils.main.RegExpUtils


m\_debug - Static variable in class uk.ac.standrews.utils.main.setutils.SupersetsManager


m\_debug - Variable in class uk.ac.standrews.utils.main.stringutils.Pool


m\_debug - Static variable in class uk.ac.standrews.utils.main.webutils.simpledomparser.XmlSearcher


m\_debug - Static variable in class uk.ac.standrews.utils.main.webutils.WebUtils


m\_initialisationIsCorrect - Variable in class uk.ac.standrews.utils.main.Initialised


m\_writer - Variable in class uk.ac.standrews.utils.main.database.Table
:   the table writer (to write data to this table)

main(String[]) - Static method in class edu.cornell.lassp.houle.RngPack.RandomApp


main(String[]) - Static method in class uk.ac.standrews.utils.main.collectionutils.setutils.SupersetsManager


main(String[]) - Static method in class uk.ac.standrews.utils.main.database.DbUtils


main(String[]) - Static method in class uk.ac.standrews.utils.main.EntrezUtils
:   Mains for testing

main(String[]) - Static method in class uk.ac.standrews.utils.main.FileUtils
:   Main method for testing

main(String[]) - Static method in class uk.ac.standrews.utils.main.guiutils.GuiUtils
:   For testing

main(String[]) - Static method in class uk.ac.standrews.utils.main.guiutils.SimpleRadioButtonPanel
:   For testing

main(String[]) - Static method in class uk.ac.standrews.utils.main.Scripts


main(String[]) - Static method in class uk.ac.standrews.utils.main.setutils.SupersetsManager


main(String[]) - Static method in class uk.ac.standrews.utils.main.Utils
:   Main method for testing

main(String[]) - Static method in class uk.ac.standrews.utils.test.AllDatabaseUtilsTests


main(String[]) - Static method in class uk.ac.standrews.utils.test.AllMapUtilsTests


main(String[]) - Static method in class uk.ac.standrews.utils.test.AllUtilsTests


main(String[]) - Static method in class uk.ac.standrews.utils.test.stats.AllStatsUtilsTests


makeDefault() - Static method in class cern.jet.random.engine.RandomEngine
:   Constructs and returns a new uniform random number engine seeded with the
    current time.

makeDefaultGenerator() - Static method in class cern.jet.random.AbstractDistribution
:   Constructs and returns a new uniform random number generation engine
    seeded with the current time.

Managed - Class in uk.ac.standrews.utils.main.database
:   A class with a manager object

Managed(Manager) - Constructor for class uk.ac.standrews.utils.main.database.Managed


Manager - Class in uk.ac.standrews.utils.main.database
:   A manager manages: for example, for a database object, the `DbManager`
    manages a `DbCreator` to create the tables, `DbReader` to read
    data from and `DbWriter` to write data to the database.

Manager() - Constructor for class uk.ac.standrews.utils.main.database.Manager


map() - Static method in class uk.ac.standrews.utils.test.maputils.TableMapTest


mapIsNullOrEmpty(Map) - Static method in class uk.ac.standrews.utils.main.maputils.MapUtils


mapIsNullOrEmpty() - Method in class uk.ac.standrews.utils.main.maputils.OneToManyMap
:   public void initialise(Vector keys, Vector values) { this.setMap();
    this.generateMap(keys, values);
    }

MapOfMaps - Class in uk.ac.standrews.utils.main.maputils
:   A map of maps.

MapOfMaps(boolean) - Constructor for class uk.ac.standrews.utils.main.maputils.MapOfMaps


MapOfMaps(boolean, int, int, int) - Constructor for class uk.ac.standrews.utils.main.maputils.MapOfMaps


MapOfMaps(boolean, Vector<String>, Vector<String>, Vector<String>) - Constructor for class uk.ac.standrews.utils.main.maputils.MapOfMaps


MapOfMaps(boolean, Vector<String>, Vector<String>, Vector<String>, int, int, int) - Constructor for class uk.ac.standrews.utils.main.maputils.MapOfMaps


MapOfMapsTest - Class in uk.ac.standrews.utils.test.maputils


MapOfMapsTest() - Constructor for class uk.ac.standrews.utils.test.maputils.MapOfMapsTest


mapToString(Map) - Static method in class uk.ac.standrews.utils.main.maputils.MapUtils
:   Creates a string representation of a map, as index,tab,key,tab,value

MapUtils - Class in uk.ac.standrews.utils.main.maputils
:   Utilities to manipulate maps and hashmaps

MapUtils() - Constructor for class uk.ac.standrews.utils.main.maputils.MapUtils


MapUtilsTest - Class in uk.ac.standrews.utils.test.maputils
:   Class

MapUtilsTest() - Constructor for class uk.ac.standrews.utils.test.maputils.MapUtilsTest


MAX\_BIGINT\_LENGTH - Static variable in class uk.ac.standrews.utils.main.database.SqlUtils
:   Maximum length in characters for a signed SQL INT BIGINT[(M)] A large
    integer.

MAX\_CHAR\_LENGTH - Static variable in class uk.ac.standrews.utils.main.database.SqlUtils
:   Maximum length in characters for an SQL CHAR column CHAR(M) A
    fixed-length string.

MAX\_FILE\_SIZE - Static variable in class uk.ac.standrews.utils.main.database.DbUtils
:   maximum size of file that can be stored in database field text ( 1,000,000 bytes ~= 1 Mb)

MAX\_INT\_LENGTH - Static variable in class uk.ac.standrews.utils.main.database.SqlUtils
:   Maximum length in characters for a signed SQL INT INT[(M)] A normal-size
    integer.

maxlev - Static variable in class edu.cornell.lassp.houle.RngPack.Ranlux
:   Maximum luxury level: `maxlev=4`

md5Hash(String) - Static method in class uk.ac.standrews.utils.main.webutils.WebUtils
:   Create the md5 hash of a text From
    http://snippets.dzone.com/posts/show/3686

MersenneTwister - Class in cern.jet.random.engine
:   MersenneTwister (MT19937) is one of the strongest uniform pseudo-random
    number generators known so far; at the same time it is quick.

MersenneTwister() - Constructor for class cern.jet.random.engine.MersenneTwister
:   Constructs and returns a random number generator with a default seed,
    which is a **constant**.

MersenneTwister(Date) - Constructor for class cern.jet.random.engine.MersenneTwister
:   Constructs and returns a random number generator seeded with the given
    date.

MersenneTwister(int) - Constructor for class cern.jet.random.engine.MersenneTwister
:   Constructs and returns a random number generator with the given seed.

messagePopUp(String, String) - Static method in class uk.ac.standrews.utils.main.guiutils.GuiUtils
:   Pop-up window with message (icon: information > "i")

MILLISECONDS - Static variable in class uk.ac.standrews.utils.main.TimeUtils


MINUTE - Static variable in class uk.ac.standrews.utils.main.TimeUtils


MONTH - Static variable in class uk.ac.standrews.utils.main.TimeUtils


multiOptionsPopUp(String, String, String[]) - Static method in class uk.ac.standrews.utils.main.guiutils.GuiUtils
:   A multiple options popup.

A B C D E F G H I J K L M N O P Q R S T U V W X Y

- Overview
- Package
- Class
- Use
- Tree
- Deprecated
- Index
- Help

- Prev Letter
- Next Letter

- Frames
- No Frames

- All Classes
